# Thermodynamics and Stability of Rhabdophanes, Hydrated Rare Earth Phosphates REPO_4_ · n H_2_O

**DOI:** 10.3389/fchem.2018.00604

**Published:** 2018-12-17

**Authors:** Anna Shelyug, Adel Mesbah, Stéphanie Szenknect, Nicolas Clavier, Nicolas Dacheux, Alexandra Navrotsky

**Affiliations:** ^1^Peter A. Rock Thermochemistry Laboratory and NEAT ORU, University of California, Davis, Davis, CA, United States; ^2^ICSM, CEA, CNRS, ENSCM, Univ Montpellier, Bagnols sur Cèze, France

**Keywords:** lanthanides, rhabdophanes, monazites, enthalpy, entropy, free energy of formation, stability

## Abstract

Rare earth phosphates comprise a large family of compounds proposed as possible nuclear waste disposal forms. We report structural and thermodynamic properties of a series of rare earth rhabdophanes and monazites. The water content of the rhabdophanes, including both adsorbed and structural water, decreases linearly with increase in ionic radius of the rare earth. The energetics of the transformation of rhabdophane to monazite plus water and the enthalpy of formation of rhabdophane from the constituent oxides was determined by high temperature drop solution calorimetry. The former varies linearly with the ionic radius of the lanthanide, except for cerium. By combining the enthalpy of formation determined by high temperature drop solution calorimetry and the free energy of formation determined previously by solubility experiments, a complete set of thermodynamic data was derived for the rhabdophanes. They are thermodynamically metastable with respect to the corresponding monazites plus water at all temperatures under ambient pressure conditions. This conclusion strengthens the case for monazites being an excellent nuclear waste form.

## Introduction

Rare earth orthophosphates (REPO_4_ · n H_2_O, where RE is a rare earth element, i.e., lanthanide plus yttrium and scandium) are widespread minerals. Their hydrated forms are commonly known as rhabdophane (Mooney, 1948, 1950; Mesbah et al., 2014) and churchite (Kohlmann et al., 1994), while their anhydrous forms are monazite (Clavier et al., 2011) and xenotime (Ni et al., 1995). These phosphate minerals are a primary source of rare earths and thorium. They also contain significant amounts of uranium (McCarthy et al., 1980; Bregiroux et al., 2007). There is strong interest in monazites for geochronology (Schärer, 1984; Schärer et al., 1986; Gibson and Ireland, 1995; Bowring et al., 1998), as they are able to deliver a wide range of information through the isotopic dating of rocks based on the U-Th-Pb natural radioactive decay chain due to their very high chemical durability in weathering conditions. Moreover, monazites usually remain crystalline even after very long times (i.e., geological times of billions of years) of exposure to self-irradiation by their U and Th contents. This radiation resistance makes monazite ceramics promising candidates for the specific immobilization of tetravalent and trivalent actinides coming from the reprocessing of spent nuclear fuel or from the management of plutonium (Ewing, 1999).

From a structural point of view, phosphate materials containing light RE ranging from La to Gd form anhydrous monazite (REPO_4_, monoclinic, P2_1_/n) or hydrated rhabdophane (REPO_4_ · n H_2_O) (Mesbah et al., 2014). For heavier RE (Gd to Lu) plus Y, anhydrous xenotime (tetragonal, I4_1_/amd) is formed. Another hydrated phosphate is reported under the name of churchite (Assaoudi and Ennaciri, 1997) which crystallizes in the gypsum structure type in the monoclinic C_2_/c space group. Three lanthanide elements (Gd, Tb, Dy) are able to form all four phosphate varieties (Boatner, 2002), which makes the preparation of single phase materials both very exciting and rather challenging based on the complex relationships among the four forms.

In the immobilization of actinides, a strong interplay between rhabdophane and monazite is often seen. Indeed, rhabdophane is a very convenient synthetic precursor for monazite when using wet chemistry methods (Terra et al., 2003; Du Fou de Kerdaniel et al., 2007; Clavier et al., 2011). Due to their very low aqueous solubility, rhabdophanes are often precipitated from aqueous solution with quantitative recovery yields (Dacheux et al., 2013; Gausse et al., 2016). Additionally, rhabdophanes are formed during the dissolution of monazites in various experimental conditions when reaching saturation conditions in solution (Clavier et al., 2006; Du Fou de Kerdaniel et al., 2007; Gausse et al., 2016, 2018). Usually, rhabdophanes are precipitated at lower temperatures than monazites. Thus, rhabdophanes often act as sequestration phases for lanthanide and actinide elements, enhancing the very high chemical durability of monazites during dissolution and leaching.

Despite this broad interest in rhabdophane materials and their relation to the anhydrous monazite and xenotime phosphate polymorphs, there is very little information about their thermodynamic stability. Especially, the energetics of the transformation of rhabdophane to monazite needs to be investigated in order to constrain the mechanism and the temperature of transition. Thus, the aim of this paper is to determine the thermodynamic properties of rhabdophane phases and to compare their stability to that of monazites.

## Experimental Methods

### Material Preparation

Rhabdophane samples REPO_4_ · n H_2_O (RE = La to Gd) and the corresponding monazites used in this study were the same materials synthesized and used in the previous work (Mesbah et al., 2017). The following reactants, all purchased from Sigma Aldrich and of analytical grade, had been used for the synthesis of the different rhabdophane compounds: LaCl_3_ · 7 H_2_O (99.9 %), CeCl_3_ · 7 H_2_O (99.9 %), PrCl_3_ · n H_2_O (99.9 %), NdCl_3_ · 6 H_2_O (99.9 %), SmCl_3_ · 6 H_2_O (99.9 %), EuCl_3_ · 6 H_2_O (99.9 %), and GdCl_3_ · 6 H_2_O (99 %). The strongly hygroscopic character of such salts makes difficult any accurate weighing of the solids. For this reason, stock solutions were prepared for all lanthanide elements by dissolving the corresponding salts in 1 mol L^–1^ HCl. These solutions were analyzed by ICP-OES giving RE concentrations ranging from 0.5 to 1 mol L^–1^. In addition, 15 mol L^–1^ H_3_PO_4_ (85% Normapur) was used as the source of the phosphate anions.

Rhabdophane samples REPO_4_ · n H_2_O (RE = La to Gd) were synthesized by mixing 4 mmol of lanthanide chloride with 5 mol L^−1^ H_3_PO_4_ solution, obtained by dilution of the concentrated stock solution to produce a RE:PO_4_ molar ratio of 1:1.03. The mixtures were stirred for 15 min at 60°C, transferred into a Teflon container and placed in an oven for 2 weeks at 90°C. The formed powders were then washed twice with deionized water followed by ethanol and then separated from the solution by centrifugation. Finally, they were dried at room temperature overnight in air. Each monazite sample was obtained by thermal conversion of its rhabdophane analog by heating 200 mg of each synthesized precursor at 1,100°C for 6 h in air.

Both sets of samples (rhabdophanes and monazites were stored in airtight containers at room temperature the period of approximately a year separating synthesis and calorimetry.

### Powder X-ray Diffraction

The rhabdophane and monazite powders were analyzed by powder X-ray diffraction (PXRD) using a Bruker D8 Advance diffractometer equipped with copper radiation (Cu Kα_1, 2_, λ = 1.54184 Å) and using reflection geometry in a parallel mode. All the powder patterns were collected between 5 and 120° (2θ) with a total counting time of about 3 h. Additionally, a powder pattern of pure silicon was collected in similar conditions and was used as a standard to extract the instrumental function. The resulting data were refined by the Rietveld method with the use of the Fullprof_Suite package (Rodríguez-Carvajal, 1993). During the refinement, different profile and structure parameters were allowed to vary. An anisotropic size model was added for each phase to simulate microstructural effects. The PXRD patterns corresponding to the synthesized compounds were refined in the rhabdophane structure type (LnPO_4_ · 0.667 H_2_O) crystallizing in the monoclinic C2 space group (Mesbah et al., 2014). In the same manner, the PXRD patterns obtained after the thermal treatment were refined in the monazite structure type (monoclinic system in the P2_1_/n space group).

### Thermodynamic Measurements and Data Analysis

Water content was obtained by mass loss evaluation after thermogravimetric analysis (TGA) coupled with differential scanning calorimetry (DSC) experiments on a Netzsch STA 449C. The instrument was calibrated using sapphire heat capacity measurement. 15–20 mg of powder was heated in air at 10°C min^−1^ from 30 to 1,000°C to fully eliminate water and convert the rhabdophane into monazite. The correction was performed by a “blank run” with an empty crucible prior to the experiment.

High temperature drop solution calorimetry was performed using a custom-built Tian-Calvet twin calorimeter (Navrotsky, 1977, 1997, 2014). Molten sodium molybdate (3Na_2_O - 4MoO_3_) solvent at 700°C (for all lanthanides except praseodymium) and lead borate (2PbO - B_2_O_3_) at 800°C (for praseodymium) were used. Approximately 5 mg of sample was hand-pressed into a pellet to drop from room temperature into 20 g of molten solvent. Oxygen was bubbled (5 mL min^−1^) through the solvent and flushed (70 mL min^−1^) through the gas space above it to enhance dissolution and maintain an oxidizing atmosphere. During the calorimetric experiment, any H_2_O evolved into the gas phase and was removed from the calorimeter, and the sample was dissolved to form a dilute solution of rare earth oxide and P_2_O_5_ in the calorimetric solvent. The measured signal of heat flow over time was integrated using CALISTO (Setaram) software to calculate the enthalpy of drop solution. The methodology is essentially the same than that used in earlier studies of RE phosphates and related materials (Hirsch et al., 2017; Neumeier et al., 2017).

## Results and Discussion

### Structural Description of the Samples

**Figure 1:**
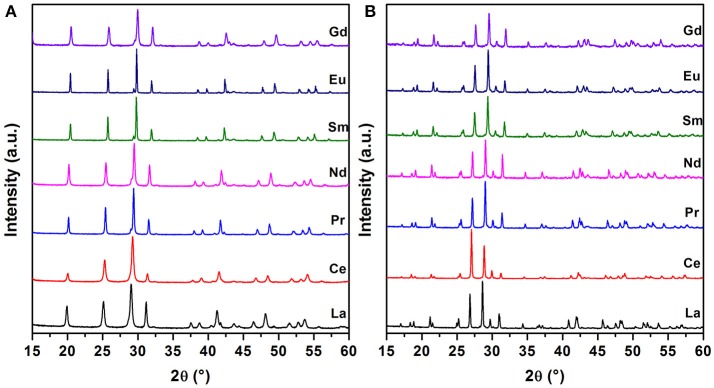
PXRD patterns of the rhabdophane **(A)** and monazite **(B)** series with RE = La to Gd.

**Figure 2:**
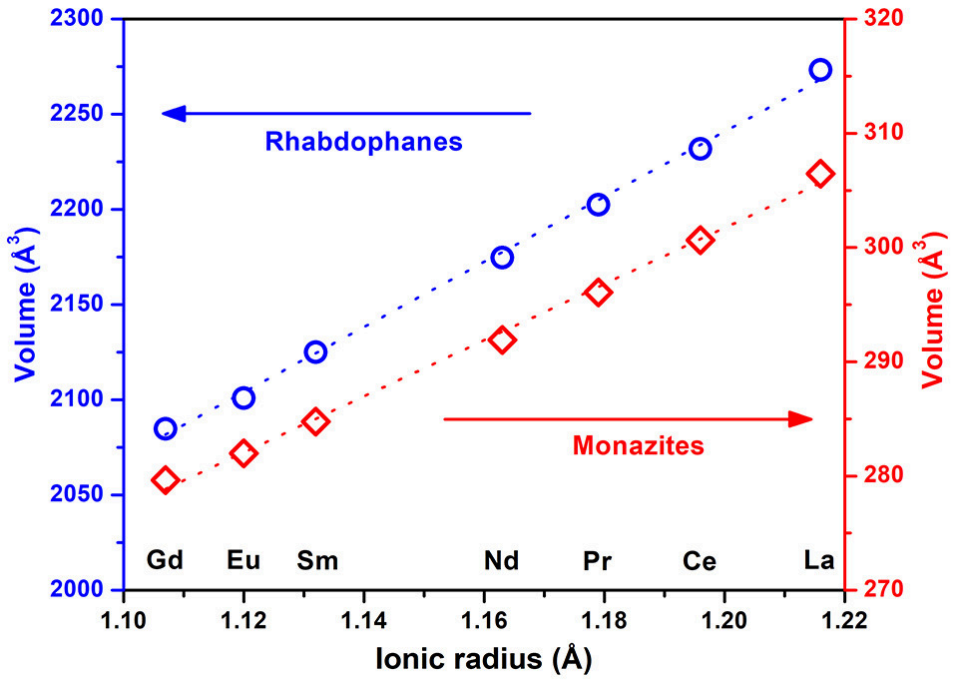
Variation of the unit cell volume vs. the ionic radius of the lanthanide element in rhabdophanes (circles) and monazites (rhombs).

Analysis of the PXRD patterns (Figure 1A) confirms that all the synthesized samples were single phases crystallizing with the rhabdophane structure type (Mesbah et al., 2014, 2017). Thermal conversion of the samples over 1,000°C always led to the formation of pure monazite compounds (Figure 1B). For all samples, the refined unit cell parameters from Rietveld refinement are gathered in Table 1 and the variation of the unit cell volumes of the rhabdophane and monazite structures are depicted in Figure 2.

**Table 1:** Unit-cell parameters of rhabdophane and monazite samples.

**RE**	**a (Å)**	**b (Å)**	**c (Å)**	**β (^**°**^)**	**Volume (Å^**3**^)**
**RHABDOPHANES**					
LaPO_4_ · 0.804 H_2_O	28.7345 (1)	7.0928 (3)	12.3351 (5)	115.27 (1)	2273.3 (2)
CePO_4_ · 0.732 H_2_O	28.6070 (2)	7.0703 (4)	12.2118 (6)	115.36 (1)	2231.8 (2)
PrPO_4_ · 0.709 H_2_O	28.4303 (7)	7.0332 (2)	12.1832 (3)	115.30 (1)	2202.4 (1)
NdPO_4_ · 0.746 H_2_O	28.2882 (1)	7.0048 (3)	12.1380 (5)	115.28 (1)	2174.7 (1)
SmPO_4_ · 0.636 H_2_O	28.0969 (8)	6.9479 (2)	12.0332 (3)	115.23 (1)	2125.0 (1)
EuPO_4_ · 0.555 H_2_O	28.0108 (5)	6.9194 (9)	11.9803 (2)	115.21 (1)	2100.9 (1)
GdPO_4_ · 0.533 H_2_O	27.9302 (8)	6.9033 (4)	11.9470 (6)	115.18 (1)	2084.6 (2)
**MONAZITES**					
LaPO_4_	6.8387 (1)	7.0752 (1)	6.5080 (1)	103.27 (1)	306.5 (1)
CePO_4_	6.7991 (2)	7.0249 (2)	6.4729 (4)	103.48 (1)	300.6 (1)
PrPO_4_	6.7679 (1)	6.9877 (1)	6.4401 (1)	103.57 (1)	296.1 (1)
NdPO_4_	6.7404 (1)	6.9557 (1)	6.4079 (1)	103.68 (1)	292.0 (1)
SmPO_4_	6.6868 (1)	6.8903 (2)	6.3667 (1)	103.88 (1)	284.8 (1)
EuPO_4_	6.6667 (1)	6.8643 (1)	6.3490 (1)	103.95 (1)	282.0 (1)
GdPO_4_	6.6497 (1)	6.8447 (2)	6.3322 (2)	104.01 (1)	279.6 (1)

The unit cell volume of rhabdophanes evolves linearly with increase of the ionic radius from La to Gd, which is in agreement with earlier observations (Mesbah et al., 2014, 2017). Indeed, the unit cell volume decreases when heavier elements are incorporated, reflecting the variation of the ionic radius along the lanthanide series (i.e., contraction of the 4f orbitals). (Shannon, 1976). Similar behavior is also observed for the anhydrous monazite, as shown in Figure 2.

### Dehydration Processes and Transformation of Rhabdophane to Monazite

**Figure 3:**
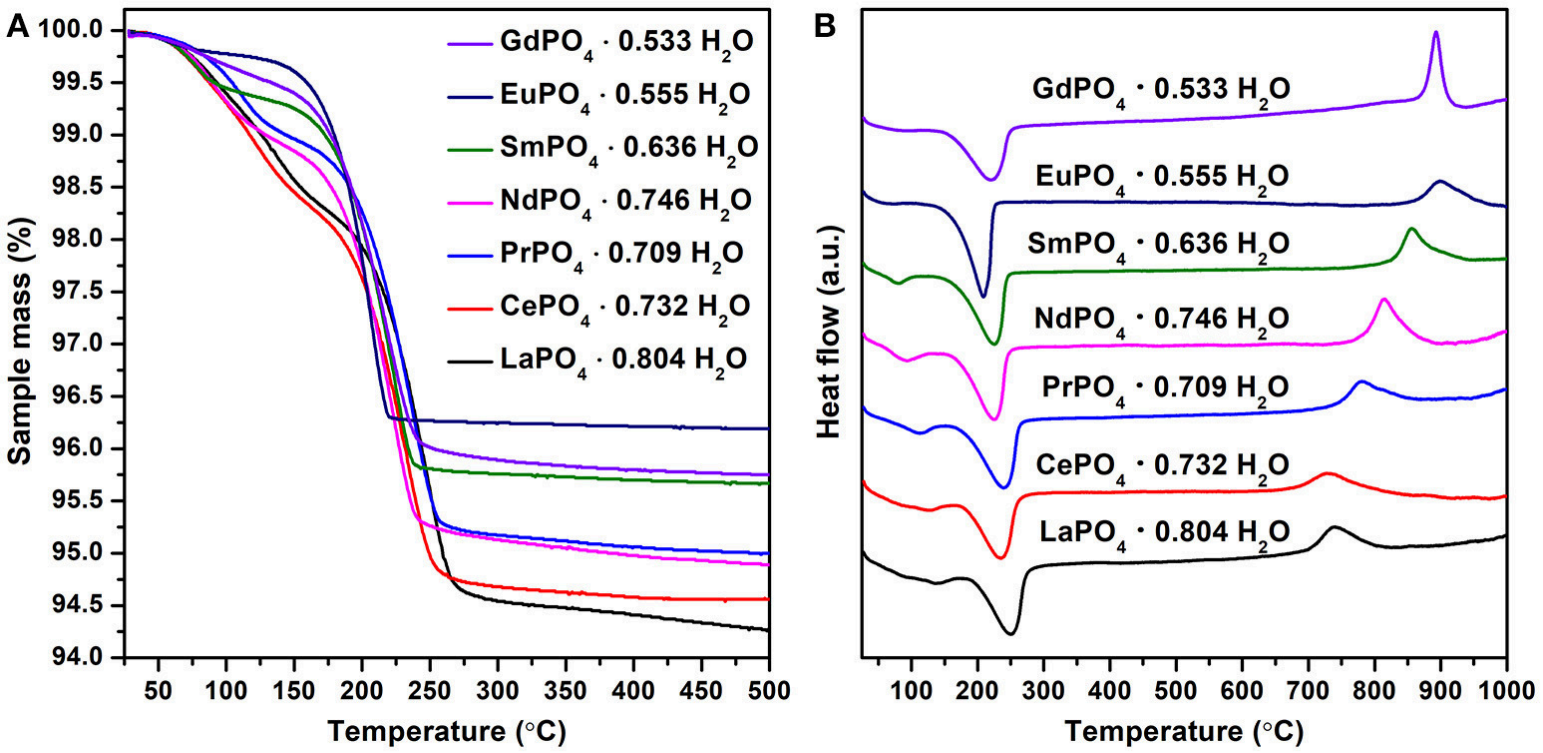
TG **(A)** and DSC **(B)** scans of rhabdophane samples.

Prior to further thermodynamic investigations all samples were studied by TG-DSC. This allowed understanding the dehydration process and the rhabdophane - monazite transition. It also confirmed the exact degree of hydration just prior to calorimetry, which is needed for the calculation of enthalpies of formation. All samples show several steps of dehydration as endothermic processes leading to dehydrated rhabdophane structure, which then exothermically transforms into monazite structure at higher temperatures (see TG and DSC data in Figure 3).

**Figure 4:**
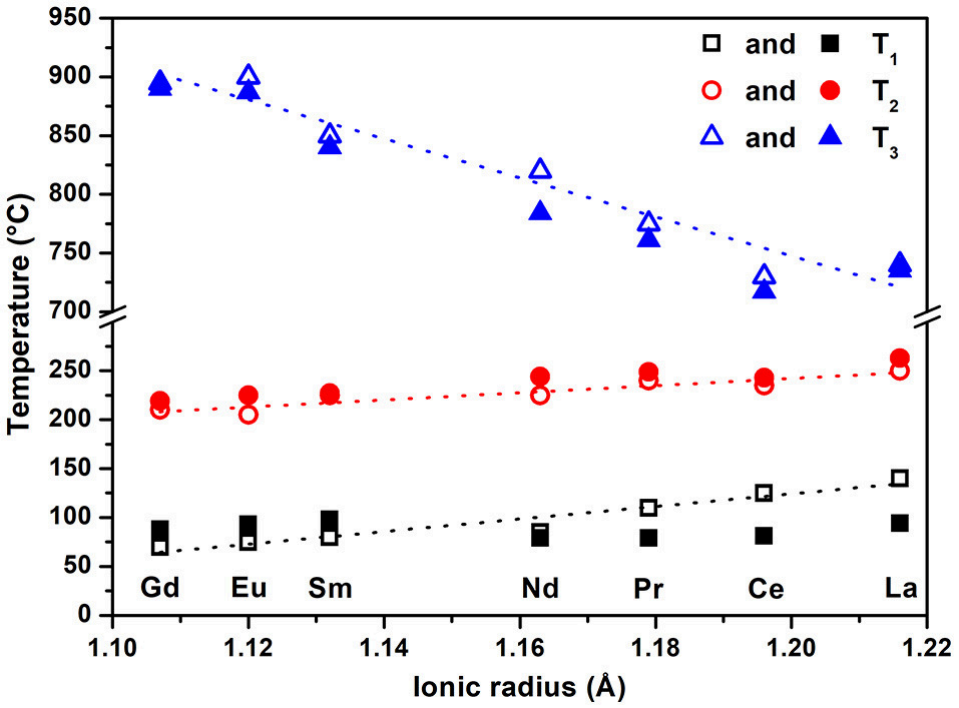
Variation of the temperatures of dehydration of rhabdophanes (T_1_ and T_2_) and transformation to monazite (T_3_) determined by TGA (open symbols) and from literature (filled symbols) (Kijkowska, 2003) as a function of ionic radius of lanthanide.

As shown in Table 2, both water contents and dehydration schemes were found to vary depending on the lanthanide element considered. Generally, there is a linear increase in temperature of dehydration and transition with respect to ionic radius (Figure 4). The measured water content ranged from 0.804 (La) to 0.533 (Gd) in the samples prepared during this work. Previous studies (Mesbah et al., 2014) report similar values ranging from 0.6 to 1.0 mole of water. This variation could come from the modification of the size of the channels in the rhabdophane structure when larger lanthanides are present that are able to have more water molecules in their coordination sphere. Due to the possibility of the intermediate hydrated compounds discussed in the abovementioned work, at least two distinct steps between ambient temperature and 250°C can be noted, first leading to the stabilization of LnPO_4_ · 0.5 H_2_O phase (monoclinic, C2) then finally to the anhydrous form LnPO_4_ (hexagonal, P3_1_21) as follows:

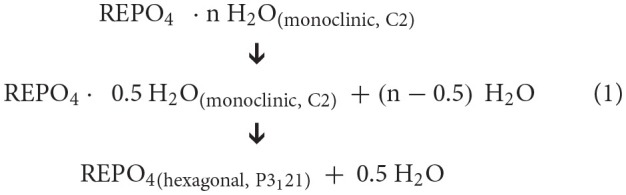



**Table 2:** Water content (n), enthalpy of dehydration **(ΔH**_**dehydr**_**)**, transition **(ΔH**_**trans**_**)** and reaction **(ΔH**_**react**_**)** of rhabdophane to monazite plus water estimated from DSC and high temperature drop solution calorimetry.

**RE**	**Water content** **(*n*)**	**ΔH_dehydr_** **kJ mol^**−1**^ of sample**	**ΔH_dehydr_** **kJ mol^**−1**^ of water**	**ΔH_trans_** **kJ mol^**−1**^ of sample**	**Δ**$\Delta H_{react}^{DSC}$ **kJ mol**^**−1**^	**Δ**$\Delta H_{react}^{ox-melt\;}$ **kJ mol**^**−1**^
La	0.804	59.1	73.5	−14.4	9.3 ± 5	−3.19 ± 1.58
Ce	0.732	50.0	68.3	−15.9	1.9 ± 5	16.79 ± 1.18
Pr	0.709	52.6	74.1	−15.2	6.1 ± 5	−4.57 ± 2.99
Nd	0.746	50.2	67.2	−23.1	−5.7 ± 5	0.82 ± 1.80
Sm	0.636	46.9	73.8	−18.5	0.4 ± 5	9.36 ± 1.61
Eu	0.555	43.9	79.2	−16.6	2.9 ± 5	13.25 ± 1.47
Gd	0.533	49.5	86.1	−21.2	1.3 ± 5	10.65 ± 1.33
	Average:	50.3 ± 1.4^*^	74.6 ± 1.8^*^			

* *Error is calculated as two standard deviations of the mean*.

All intermediate products were irreversibly converted to monazites at temperatures ranging from 700 to 950°C. Such transformation was associated with an exothermic peak on all DTA curves. The variation of the temperature associated with the rhabdophane → monazite transition is shown as a function of the ionic radius in Figure 4. It is in a good agreement with the data previously reported (Kijkowska, 2003), showing a decrease with an increase of lanthanide ionic radius. However, since the transformation was not reversed, it is unknown whether the observed temperature represents an equilibrium reaction. Considering possible differences in synthesis procedures among these studies and error in temperature determination, reported differences as high as ±10°C are not surprising. The general agreement between current and prior temperatures suggests that, even if kinetically controlled, the dehydration process appears similar in several separately prepared materials.

The enthalpy of dehydration was found as the heat absorbed during water loss from DSC by integration of the peaks associated with water loss. This process occurs at the temperature of dehydration T_2_ and represents the reaction:

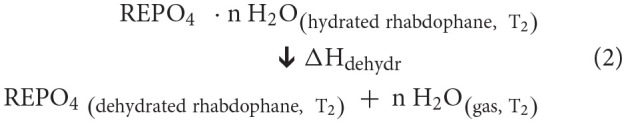



The integrations were performed across both steps and recalculated per mole of sample and per mole of water in order to obtain the enthalpies of dehydration (ΔH_dehydr_) (given in Table 2). The dehydration enthalpies are relatively constant with an average value of 50.3 ± 1.8 kJ mol^−1^ of RE and 74.6 ± 1.8 kJ mol^−1^ of water.

Enthalpy of transition was obtained by integration of the exothermic peak at T_3_. This process corresponds to the following reaction:

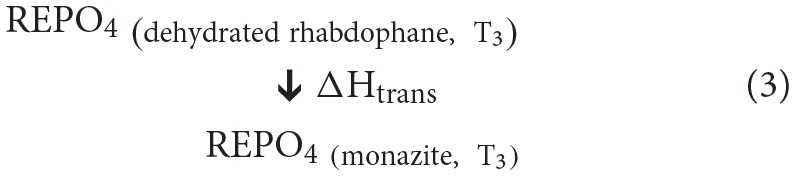



Enthalpy of reaction of hydrated rhabdophane to monazite plus water was estimated from DSC measurements using the following relation:

(4) $$\Delta {\text{H}}_{\text{react }}^{\text{DSC}}=\Delta {\text{H}}_{\text{dehydr}}-\Delta {\text{H}}^{{}^{\circ}}{}_{\text{vapor}}\times \text{n}+\Delta {\text{H}}_{\text{trans}}$$

where $\Delta {\text{H}}_{\text{vapor}}^{\circ }$ = 44 kJ mol^−1^ is standard enthalpy of vaporization of water at 25°C.

The enthalpy of reaction ($\Delta {\text{H}}_{\text{react}}^{\text{ox - melt}}$) which is associated with the reaction from rhabdophane to monazite plus liquid water at room temperature was obtained by high temperature drop solution calorimetry.

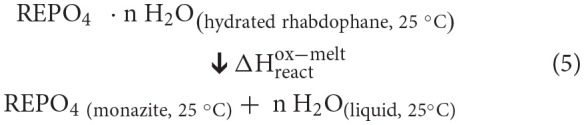



The enthalpy associated with this transformation was calculated from drop solution enthalpy using the following equation:

(6) $$\begin{array}{r} {\Delta \text{H}}_{\text{react}}^{\text{ox}-\text{melt}}={\Delta \text{H}}_{\text{ds},700}^{\text{rhabd}}-{\Delta \text{H}}_{\text{ds},700}^{\text{monaz}}-\text{ n }{\Delta \text{H}}_{25-700}^{\text{water}} \end{array}$$

where $\Delta {\text{H}}_{\text{ds,700}}^{\text{rhabd}}$ and $\Delta {\text{H}}_{\text{ds},700}^{\text{monaz}}$ stand for enthalpy of drop solution of rhabdophane and monazite in sodium molybdate at 700°C and $\Delta {\text{H}}_{25-700}^{\text{water}}$ is heat uptake by water when it is heated inside the calorimeter from 25 to 700°C (this reaction and overall thermodynamic data from high temperature drop solution calorimetry experiments will be described in more details in the following sections).

**Figure 5:**
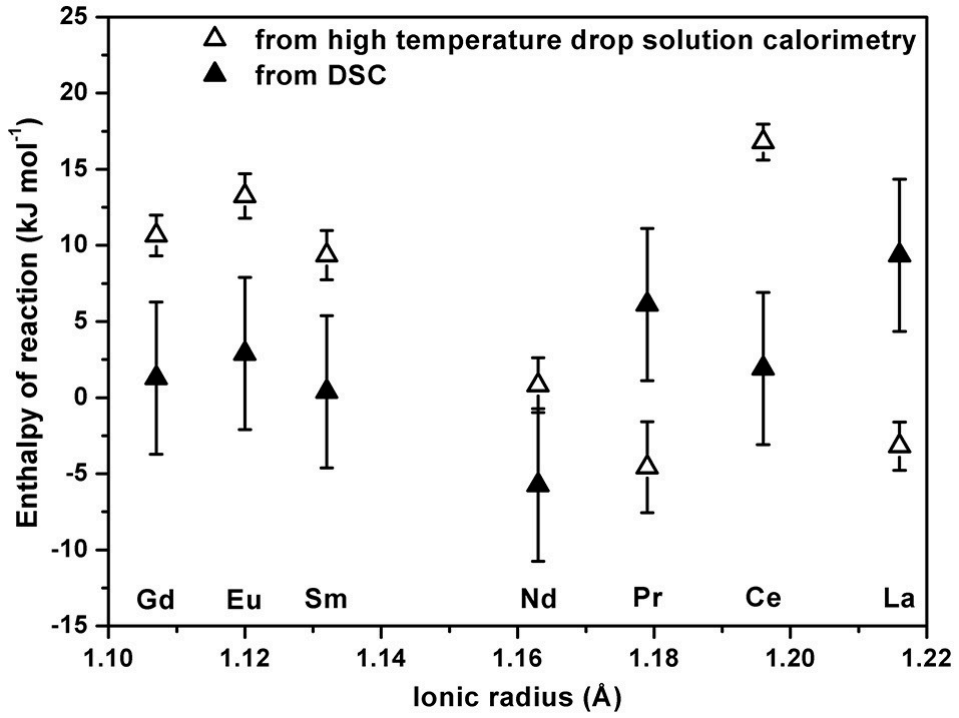
Enthalpy of reaction of rhabdophane to monazite plus water as a function of ionic radius of lanthanide calculated from drop solution enthalpy and estimated from DSC.

The values calculated from DSC data are listed in Table 2, which also gives values from high temperature drop solution calorimetry. The enthalpies of reaction associated with the transformation of rhabdophane to monazite plus water are also plotted in Figure 5.

Enthalpy of reaction calculated from drop solution enthalpy becomes slightly less endothermic with increasing RE ionic radius except for the cerium sample, which deviates from the general trend for reasons that remain unclear. Since both Ce-bearing monazite and rhabdophane appear to contain only trivalent cerium, difference in oxidation state is not expected to be involved during the transformation. One of the possible reasons for the anomalous behavior of cerium may be that Ce-containing samples have more complex surface structures (Takita et al., 1998; Huang and Fabris, 2007) that could affect the energetics. The values estimated from DSC scans slightly deviate from those found by high temperature drop solution calorimetry, but this might be the consequence of estimations and the integration errors which accompany DSC experiments. Nevertheless, overall values are in reasonable agreement from both experiments and are close to zero. The data suggest that rhabdophane is energetically metastable or at best only marginally stable with respect to monazite plus water. As noted below, similar conclusions can be drawn about the free energy of the reaction, implying thermodynamic metastability. The temperatures of dehydration and final transformation listed in Table 2 probably reflect kinetic rather than thermodynamic control. The dehydrated rhabdophanes are energetically less stable than the initial fully hydrated forms. These observations, including the variable water contents, are consistent with relatively loose binding of water in the rhabdophane structure.

### Heats of Formation

**Figure 6:**
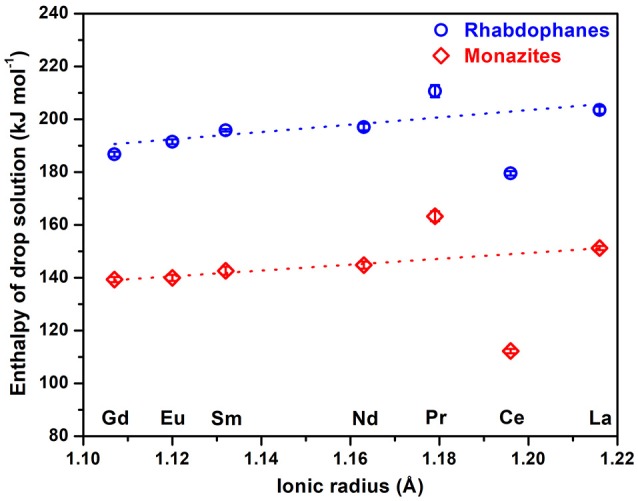
Enthalpy of drop solution of rhabdophanes (circles) and monazites (rhombs) as a function of ionic radius.

The values of drop solution enthalpy (Figure 6) increase linearly with ionic radii for both sets of materials except for Pr and Ce containing samples. It was discussed previously (Hirsch et al., 2017) that praseodymium phosphates do not dissolve well at 700°C in sodium molybdate, which results in more endothermic values for the drop solution enthalpy we observed. More exothermic values of drop solution enthalpy of cerium phosphates are the result of cerium oxidation (Ce^3+^ → Ce^4+^) in the solvent. This effect for monazite CePO_4_ and also the observed linear trend were discussed previously (Ushakov et al., 2001).

Thermodynamic cycles (see Supporting info Tables A1a–c) were built to calculate standard enthalpies of formation of rhabdophanes and monazites from oxides and elements. Complex nature of Ce and Pr samples in the solvent was assessed in separate cycles. Ushakov et al. (2001) published a thermodynamic cycle for PrPO_4_ monazite using the assumption that Pr_2_O_3_ oxidizes to Pr_6_O_11_ in sodium molybdate solvent at 700°C. However, this was not based on convincing evidence but rather on the observation that Pr_2_O_3_ does not dissolve well while Pr_6_O_11_ appears to dissolve rapidly. In order to avoid this uncertainty, a thermodynamic cycle based on the dissolution of Pr_2_O_3_ in lead borate at 800°C (where it dissolves rapidly) was used here to calculate the enthalpy of formation for both monazite and rhabdophane of praseodymium. All the measured and calculated values are given in Table 3.

**Table 3:** Enthalpy of drop solution (ΔH_ds_), formation from oxides (${\Delta \text{H}}_{\text{f},\text{ox}}^{{}^{\circ}}$), and elements (${\Delta \text{H}}_{\text{f},\text{el}}^{{}^{\circ}}$) at 25°C for rhabdophanes and monazites.

**RE**	**ΔH_ds_$\left(700^{{}^{\circ}}\text{C}\right)$**	**ΔH°_f, ox_**	**ΔH°**${}_{\text{f},e\text{l}}^{}$
	**kJ mol^**−1**^**	**kJ mol^**−1**^**	**kJ mol^**−1**^**
**RHABDOPHANES**			
LaPO_4_ · 0.804 H_2_O	203.53 ± 1.35 (9)	−342.92 ± 4.29	−2220.9 ± 4.5
CePO_4_ · 0.732 H_2_O	179.56 ± 0.83 (9)	−328.23 ± 7.21	−2189.7 ± 9.4
PrPO_4_ · 0.709 H_2_O	199.67 ± 0.98 (8) 210.67 ± 2.32 (5)^*^	−321.54 ± 6.05^*^	−2181.7 ± 6.4^*^
NdPO_4_ · 0.746 H_2_O	197.10 ± 1.22 (8)	−309.60 ± 4.57	−2178.7 ± 5.1
SmPO_4_ · 0.636 H_2_O	195.88 ± 0.48 (8)	−311.13 ± 4.22	−2156.8 ± 5.1
EuPO_4_ · 0.555 H_2_O	191.51 ± 0.91 (10)	−300.15 ± 4.07	−2042.4 ± 4.9
GdPO_4_ · 0.533 H_2_O	186.76 ± 0.92 (8)	−304.45 ± 3.96	−2119.1 ± 7.2
**MONAZITES**			
LaPO_4_	151.26 ± 0.82 (11)	−346.11 ± 3.37	−1994.4 ± 4.3
CePO_4_	112.28 ± 0.84 (10)	−316.25 ± 6.46	−1963.8 ± 9.4
PrPO_4_	147.57 ± 0.96 (8) 163.31 ± 1.89 (7)^*^	−326.11 ± 8.13^*^	−1983.5 ± 6.3^*^
NdPO_4_	144.80 ± 1.33 (9)	−308.78 ± 3.77	−1964.7 ± 5.1
SmPO_4_	142.66 ± 1.54 (8)	−301.77 ± 3.36	−1965.7 ± 5.3
EuPO_4_	139.98 ± 1.15 (11)	−286.90 ± 2.56	−1870.6 ± 4.9
GdPO_4_	139.33 ± 0.96 (8)	−293.80 ± 1.86	−1956.1 ± 7.2

*All errors in the table are propagated as two standard deviations of the mean; numbers in brackets are numbers of individual measurements*.

* *drop solution enthalpy in lead borate at 800°C. Enthalpies of formation of Pr-containing rhabdophane and monazite from oxides are calculated using thermodynamic cycle from high temperature drop solution experiments with lead borate at 800°C*.

**Figure 7:**
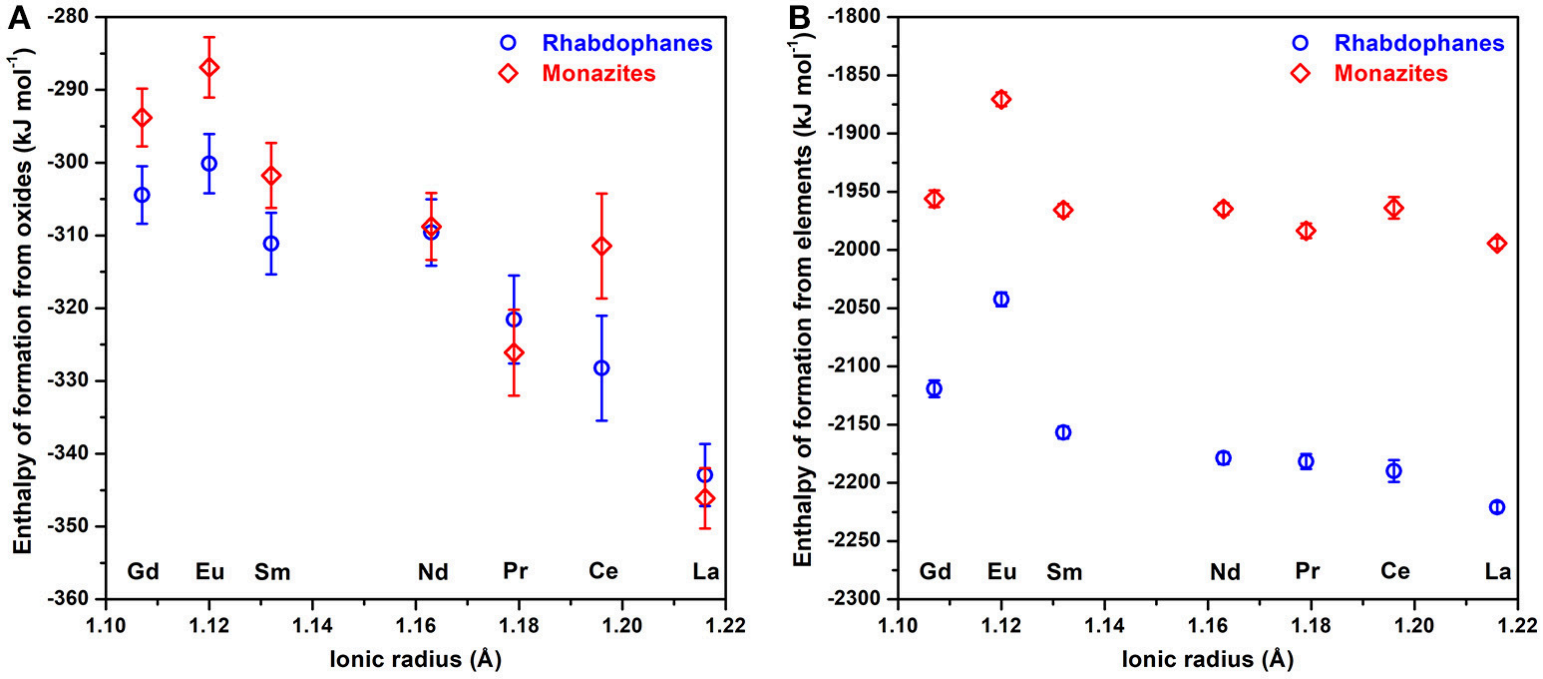
Standard enthalpy of formation at 25°C from oxides **(A)** and from elements **(B)** of rhabdophanes (circles) and monazites (rhombs).

Figure 7 shows that the standard enthalpies of formation of rhabdophanes from oxides become more exothermic with increasing RE radius, as seen previously for monazites (Ushakov et al., 2001). Neither, Pr nor Ce show anomalous behavior since we compensate for the oxidation effects in the solvent by the thermodynamic cycles. The difference in the value for La-monazite from Ushakov et al. (2001) was discussed in a recent study on La_1−x_Pr_x_PO_4_ monazite solid solutions (Hirsch et al., 2017) and was attributed to experimental problems (insufficient gas bubbling through the calorimetric solvent). Enthalpy of formation from elements for the Eu-containing samples deviates from the trend, as shown in previous work (Ushakov et al., 2001), for unknown reasons.

### Combining Solubility and Calorimetric Data for Rhabdophanes

A systematic study of the solubility of the same rhabdophane samples, LnPO_4_ · n H_2_O (Ln = La to Dy) was performed by Gausse et al. (2016) using over- and under-saturated experiments at different temperatures (25–90°C). These experiments can be used to obtain the standard Gibbs free energy of formation and, from its temperature dependence, both the enthalpy, and entropy of formation. Alternatively, the Gibbs free energy at 25°C from solubility experiments can be combined with the enthalpy of formation measured by high temperature drop solution calorimetry to obtain the entropy of formation at 25°C without relying on the temperature dependence of solubility data. This second approach is probably more accurate because the solubility data can be obtained only over a relatively small temperature range and the thermodynamic analysis of solubility data requires knowledge of or assumptions about the thermodynamic behavior of the aqueous species at higher temperatures.

The values of standard Gibbs free energy of formation from elements ($\Delta G_{\text{f},\text{el}}^{{}^{\circ}}$), of Gausse et al. (2016) were calculated considering that the water content per formula unit was 0.667 for the whole series. In the present work the water content, n, was measured by TGA and was found to vary from 0.533 (for Gd) to 0.804 (for La), thus the values from Gausse et al. (2016) were recalculated according to a new water content. These new values were used to find standard Gibbs free energy at 25°C of formation from oxides:

(7) $$\begin{array}{l} \Delta {\text{G}}_{\text{f,ox}}^{{}^{\circ}}\left(\text{Ln}{\text{PO}}_{4}\cdot \text{n }{\text{H}}_{2}\text{O}\right)=\\ \;\;\;\;\;\Delta {\text{G}}_{\text{f,el}}^{{}^{\circ}}\left({\text{LnPO}}_{4}\cdot \text{n }{\text{H}}_{2}\text{O}\right)-\frac{1}{2}\Delta {\text{G}}_{\text{f,el}}^{{}^{\circ}}\left({\text{Ln}}_{2}{\text{O}}_{3}\right)\\ \;\;\;\;\;-\frac{1}{2}\Delta {\text{G}}_{\text{f},\text{el}}^{{}^{\circ}}\left({\text{P}}_{2}{\text{O}}_{5}\right)-\text{n}\Delta {\text{G}}_{\text{f},\text{el}}^{{}^{\circ}}\left({\text{H}}_{2}\text{O}\right) \end{array}$$

Then, the enthalpies of formation of rhabdophanes and monazites from oxides obtained from high temperature drop solution calorimetry in the previous section were used to calculate entropies of formation from oxides 25°C.

(8) $$\Delta {\text{S}}_{\;\text{f},\text{ox}}^{\circ }=\frac{\Delta {\text{H}}_{\;\text{f},\text{ox}}^{\circ }-\Delta {\text{G}}_{\;\text{f},\text{ox}}^{\circ }}{298.15}$$

**Figure 8:**
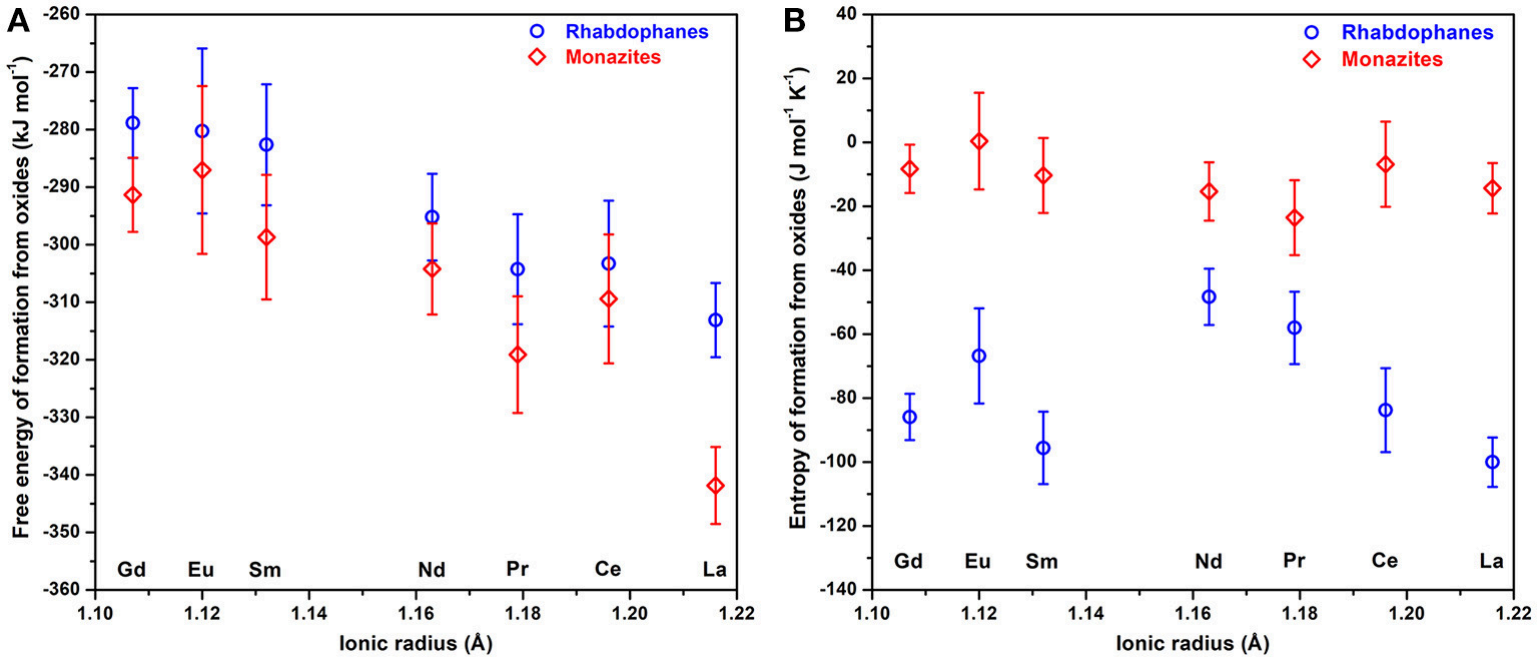
Standard Gibbs free energy of formation from oxides **(A)** and standard entropy of formation from oxides **(B)** of rhabdophanes (circles) and monazites (rhombs) as a function of ionic radius.

The values of standard free energy and entropy of formation from oxides are plotted on Figure 8.

The values of standard entropy of formation of monazites from oxides are slightly negative and the small magnitudes are reasonable for solid state reactions in which no gases are consumed or produced, while those for rhabdophanes are more negative, reflecting the confinement of liquid water.

Using standard entropies of oxides, those of rhabdophanes and monazites were calculated as

(9) $$\begin{array}{l} {\text{S}}_{\text{m}}^{{}^{\circ}}(\text{ox})\left({\text{LnPO}}_{4}\cdot \text{n }{\text{H}}_{2}\text{O}\right)=\\ \;\;\;\;\Delta {\text{S}}_{\text{f,ox}}^{{}^{\circ}}\left(\text{LnPO}{}_{4}\cdot \text{n }{\text{H}}_{2}\text{O}\right)+\frac{1}{2}{\text{S}}_{\text{m}}^{{}^{\circ}}\left({\text{Ln}}_{2}{\text{O}}_{3}\right)\\ \;\;\;\;+\frac{1}{2}{\text{S}}_{\text{m}}^{{}^{\circ}}\left({\text{P}}_{2}{\text{O}}_{5}\right)+\text{n}\times {\text{S}}_{\text{m}}^{{}^{\circ}}\left({\text{H}}_{2}{\text{O}}_{\left(\text{l}\right)}\right) \end{array}$$

**Figure 9:**
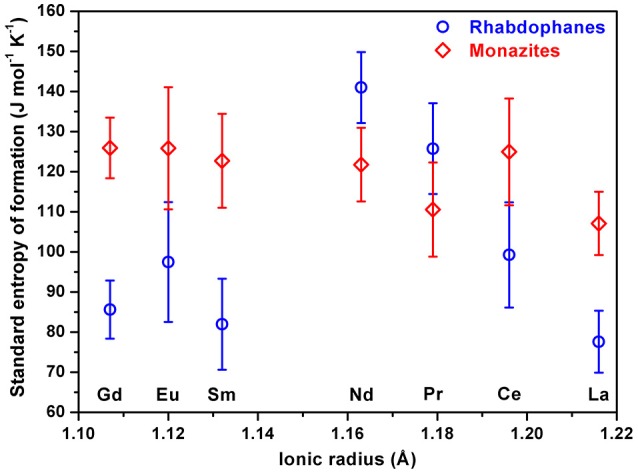
Standard molar entropy at 25°C of rhabdophanes (circles) and monazites (rhombs) calculated using equation (9) as a function of ionic radius.

These are plotted against ionic radius in Figure 9. We also calculated standard entropy from enthalpy and free energy of formation from elements to check the consistency of the data as:

(10) $$\begin{array}{l} {\text{S}}_{\text{m}}^{{}^{\circ}}(\text{el})\left({\text{LnPO}}_{4}\cdot \text{n }{\text{H}}_{2}\text{O}\right)=\\ \;\;\;\;\;\Delta {\text{S}}_{\text{f},\text{el}}^{{}^{\circ}}\left(\text{LnP}{\text{O}}_{4}\cdot \text{n }{\text{H}}_{2}\text{O}\right)+{\text{S}}_{\text{m}}^{{}^{\circ}}(\text{Ln},\text{cr})\\ \;\;\;\;\;+{\text{S}}_{\text{m}}^{{}^{\circ}}(\text{P},\text{cr})+2\times {\text{S}}_{\text{m}}^{{}^{\circ}}\left({\text{O}}_{2},\text{g}\right)+\\ \;\;\;\;\;+\text{n}\times \left[\frac{1}{2}{\text{S}}_{\text{m}}^{{}^{\circ}}\left({\text{O}}_{2},\text{g}\right)+{\text{S}}_{\text{m}}^{{}^{\circ}}\left({\text{H}}_{2},\text{g}\right)\right] \end{array}$$

All thermodynamic data used and calculated this far are compiled in Table 4. Auxiliary data (values for elements and oxides) are given in supporting info (Table A2).

**Table 4:** Thermodynamic data at 25°C calculated from solubility experiments for rhabdophanes and using data from (Navrotsky et al., 2015) for monazites. Standard molar entropies are given as calculated from enthalpy and free energy of formation from oxides **[**${\text{S}}_{\text{m}}^{{}^{\circ}}$**(ox)]** with Equation (9) and from elements **[**${\text{S}}_{\text{m}}^{{}^{\circ}}$**(el)]** with Equation (10).

	**$\Delta {\text{G}}_{\text{f},\text{el}}^{{}^{\circ}}$**,	**$\Delta {\text{G}}_{\text{f},\text{ox}}^{{}^{\circ}}$**,	**$\Delta {\text{S}}_{\text{f},\text{el}}^{{}^{\circ}}$**,	**$\Delta {\text{S}}_{\text{f},\text{ox}}^{{}^{\circ}}$**,	**$\Delta {\text{S}}_{\text{m}}^{{}^{\circ}}$(ox)**,	**$\Delta {\text{S}}_{\text{m}}^{{}^{\circ}}$(el)**,
	**J mol^**−1**^ K^**−1**^**	**J mol^**−1**^ K^**−1**^**	**J mol^**−1**^ K^**−1**^**	**J mol^**−1**^ K^**−1**^**	**J mol^**−1**^ K^**−1**^**	**J mol^**−1**^ K^**−1**^**
**RHABDOPHANES**						
LaPO_4_ · 0.804 H_2_O	−2036 ± 6	−313.1 ± 6.4	−618.7 ± 7.4	−100.2 ± 7.7	77.6 ± 7.8	77.1 ± 7.8
CePO_4_ · 0.732 H_2_O	−2012 ± 8	−303.3 ± 10.9	−594.9 ± 12.2	−83.9 ± 13.1	99.3 ± 13.1	96.7 ± 14.8
PrPO_4_ · 0.709 H_2_O	−2013 ± 8	−304.2 ± 9.6	−565.6 ± 10.2	−58.2 ± 11.3	125.7 ± 11.3	125.2 ± 11.0
NdPO_4_ · 0.746 H_2_O	−2013 ± 6	−295.2 ± 7.5	−556.7 ± 7.8	−48.5 ± 8.8	141.0 ± 8.8	139.8 ± 8.8
SmPO_4_ · 0.636 H_2_O	−1982 ± 10	−282.6 ± 10.5	−587.6 ± 11.1	−109.2 ± 11.3	82.0 ± 11.3	81.6 ± 11.3
EuPO_4_ · 0.555 H_2_O	−1869 ± 13	−280.2 ± 14.3	−580.3 ± 14.2	−77.0 ± 14.9	97.5 ± 15.0	81.3 ± 14.2
GdPO_4_ · 0.533 H_2_O	−1952 ± 5	−278.8 ± 6.1	−559.7 ± 8.7	−86.0 ± 7.2	85.6 ± 7.2	84.5 ± 8.8
**MONAZITES**						
LaPO_4_	−1875 ± 5	−341.8 ± 6.7	−401.7 ± 7.5	−14.3 ± 7.9	107.1 ± 7.9	106.6 ± 8.0
CePO_4_	−1845 ± 10	−309.4 ± 11.2	−398.5 ± 12.5	−6.8 ± 13.3	125.0 ± 13.3	122.4 ± 15.0
PrPO_4_	−1860 ± 7	−319.1 ± 10.1	−415.3 ± 10.6	−23.5 ± 11.7	110.6 ± 11.7	110.1 ± 11.4
NdPO_4_	−1845 ± 6	−304.2 ± 7.9	−401.9 ± 8.1	−15.3 ± 9.1	121.8 ± 9.2	120.6 ± 9.2
SmPO_4_	−1847 ± 6	−298.7 ± 10.8	−398.5 ± 11.5	−10.3 ± 11.7	122.7 ± 11.7	122.4 ± 11.7
EuPO_4_	−1739 ± 6	−287.0 ± 14.6	−422.5 ± 14.4	0.4 ± 15.1	125.9 ± 15.2	109.7 ± 13.8
GdPO_4_	−1838 ± 7	−291.3 ± 6.4	−395.0 ± 8.9	−8.3 ± 7.6	125.9 ± 7.6	124.8 ± 8.8

*All errors in the table are propagated as two standard deviations of the mean*.

Using these standard molar entropies and entropy of liquid water, we calculated entropies and, further, using the enthalpy of reaction estimated from high temperature drop solution calorimetry (Table 2), free energies of reaction of rhabdophane to monazite plus water:

(11) $$\begin{array}{l} \Delta {\text{S}}_{\text{react}}^{{}^{\circ}}=-{\text{S}}_{\text{m}}^{{}^{\circ}}\left({\text{LnPO}}_{4}\times \text{n }{\text{H}}_{2}\text{O}\right)\\ \;\;\;\;\;\;+{\text{S}}_{\text{m}}^{{}^{\circ}}\left({\text{LnPO}}_{4}\right)-\text{n}\times {\text{S}}_{\text{m}}^{{}^{\circ}}\left({\text{H}}_{2}{\text{O}}_{\text{l},25}\right) \end{array}$$

(12) $$\Delta {\text{G}}_{\text{react}}^{{}^{\circ}}=\Delta {\text{H}}_{\text{react}}^{\text{ox}-\text{melt}}-298.15\times \Delta {\text{S}}_{\text{react}}^{{}^{\circ}}$$

**Figure 10:**
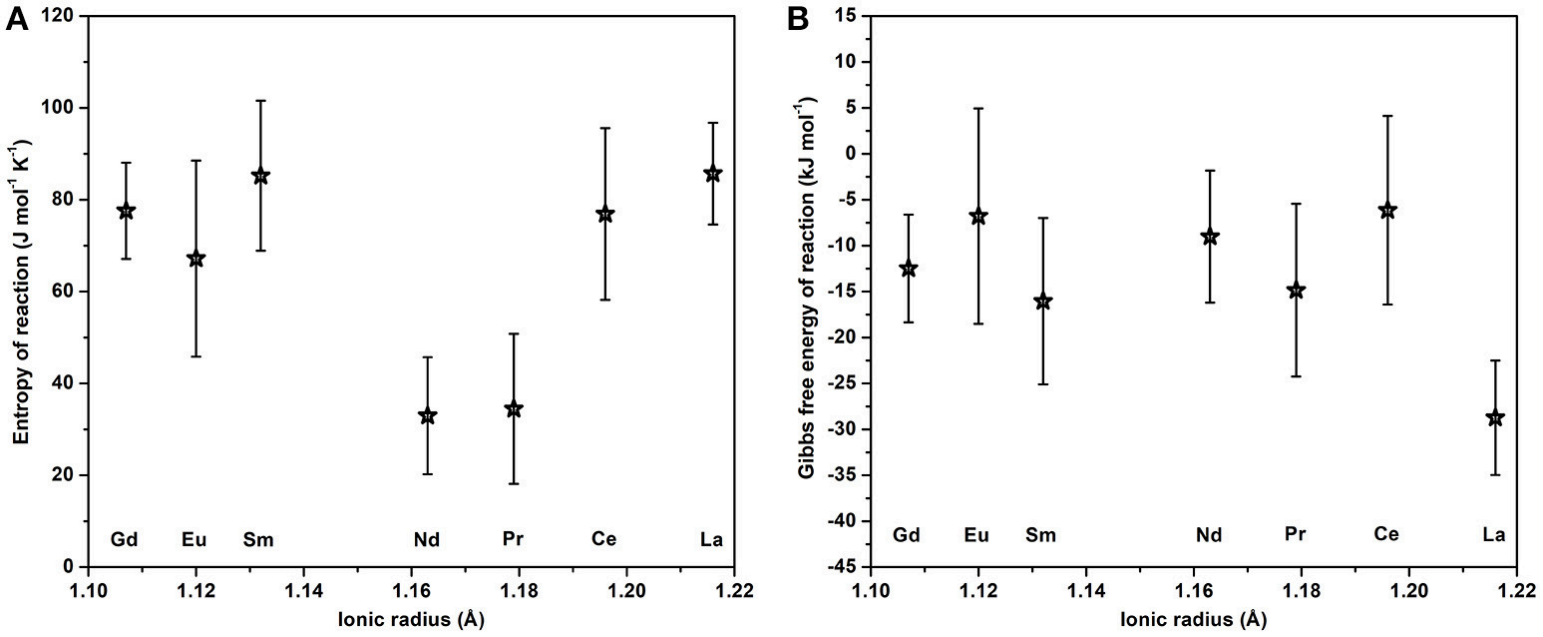
Standard entropy **(A)** and Gibbs free energy **(B)** of reaction of rhabdophane to monazite plus water at 25°C plotted against ionic radius of lanthanide element.

These values are plotted on Figure 10 and given in Table 5.

**Table 5:** Enthalpy, entropy and free energy of reactions of rhabdophane to monazite plus water transition at 25°C.

**Reaction**	**$\Delta H_{react}^{ox-melt}$**	**$\Delta S_{react}^{\circ }$**	**$\Delta G_{react}^{\circ }$**
	**kJ mol^**−1**^**	**J mol^**−1**^ K^**−1**^**	**kJ mol^**−1**^**
LaPO_4_ · 0.804 H_2_O → LaPO_4_ + 0.804 H_2_O	−3.19 ± 1.58	85.7 ± 11.1	−28.8 ± 8.5
CePO_4_ · 0.732 H_2_O → CePO_4_ + 0.732 H_2_O	16.79 ± 1.18	76.9 ± 18.7	−6.2 ± 14.4
PrPO_4_ · 0.709 H_2_O → PrPO_4_ + 0.709 H_2_O	−4.57 ± 2.99	34.5 ± 16.3	−14.9 ± 12.3
NdPO_4_ · 0.746 H_2_O → NdPO_4_ + 0.746 H_2_O	0.82 ± 1.80	33.0 ± 12.7	−9.1 ± 9.5
SmPO_4_ · 0.636 H_2_O → SmPO_4_ + 0.636 H_2_O	9.36 ± 1.61	85.2 ± 16.3	−20.1 ± 10.8
EuPO_4_ · 0.555 H_2_O → EuPO_4_ + 0.555 H_2_O	13.25 ± 1.47	67.2 ± 21.3	−9.8 ± 13.1
GdPO_4_ · 0.533 H_2_O → GdPO_4_ + 0.533 H_2_O	10.65 ± 1.33	77.6 ± 10.5	−12.5 ± 8.0

*All errors in the table are propagated as two standard deviations of the mean*.

The data above suggest that rhabdophanes are thermodynamically metastable with respect to monazite plus liquid water, even at room temperature. Mesbah et al. (2014) performed rehydration experiments on the same samples used in this study. They observed that the rhabdophane structure could reverse the dehydration. However, once samples have transformed to the monazite structure there is no rehydration or back-reaction to rhabdophane in the presence of liquid water. Similarly, rhabdophane synthesis in aqueous solution proceeds directly and often rapidly from dissolved ionic species, possibly reflecting the hydrated nature of rare earth ions. Dehydration and eventual irreversible transformation to monazite requires high temperature, apparently for kinetic rather than thermodynamic reasons since the present data suggest the metastability of rhabdophane under all conditions. We stress that once monazite is formed, it cannot transform to rhabdophane even in contact with an aqueous phase for a long time because the thermodynamic data show that monazite plus water is the slightly more thermodynamically stable phase assemblage. This finding is important for nuclear waste immobilization. In order to check this point, we contacted rhabdophane CePO_4_ · n H_2_O and monazite CePO_4_ with aqueous solution (10–1 M HNO_3_) for several weeks at 120°C and 70°C, respectively. While monazite was not affected, rhabdophane turned into monazite progressively, with transformation almost complete after 2 weeks.

## Conclusion

The enthalpies of formation of a series of rhabdophanes were measured by high temperature drop solution calorimetry and the energetics of dehydration and transformation to monazite plus water were determined by DSC. Combined with free energies from solubility measurements, the data allow the calculation of entropies of formation and standard entropies of rhabdophane phases. Rhabdophanes are metastable with respect to the corresponding monazites plus water at all temperatures under ambient pressure conditions. However, presumably due to a more rapid kinetics of precipitation, rhabdophane is often formed initially leaching of phosphate based ceramics, especially for temperatures representative of long-term repositories. This rapid formation of rhabdophane, followed by slower conversion to monazite, can contributes to the significant delay of actinide and rare earth releases in environment. Additionally, monazite, once formed, can be very effective in radionuclide confinement without any rapid back-transformation to hydrous phases, even on long time scales.

## Author Contributions

AS and SS performed experiments and calculations. AS, SS, NC, ND, AM, and AN provided theoretical explanations and edited the manuscript.

### Conflict of Interest Statement

The authors declare that the research was conducted in the absence of any commercial or financial relationships that could be construed as a potential conflict of interest.
