# Supplementary material for: Thermodynamics and Stability of Rhabdophanes, Hydrated Rare Earth Phosphates REPO4 · n H2O
Source: Front Chem. 2018 Dec 17;6:604. doi: 10.3389/fchem.2018.00604 (PMC6304437; doi:10.3389/fchem.2018.00604)
Supplement: Supplementary file 1 [file Table_1.DOCX]

Supplementary Material

Thermodynamics and stability of rhabdophanes, hydrated rare earth phosphates REPO_4_ · n H_2_O

Anna Shelyug, Adel Mesbah, Stéphanie Szenknect, Nicolas Clavier, Nicolas Dacheux and Alexandra Navrotsky*

*** Correspondence:** Alexandra Navrotsky: anavrotsky@ucdavis.edu

Table A1a. Thermodynamic cycle used for RE= Gd, Eu, Sm, Nd and La

| **Reaction** | **Enthalpy, kJ mol^-1^** |
| --- | --- |
| REPO_4_ · *n* H_2_O_(s,25)_ → 1/2 RE_2_O_3(sln,700)_ + 1/2 P_2_O_5(sln,700)_ + *n* H_2_O_(g,700)_ | **ΔH_ds_(rh, 700)** |
| REPO_4(s,25)_ → 1/2 RE_2_O_3(sln,700)_ + 1/2 P_2_O_5(sln,700)_ | **ΔH_ds_(m, 700)** |
| RE_2_O_3(s,25)_ → RE_2_O_3(sln,700)_ | **ΔH_1_ ^$^** |
| P_2_O_5(s,25)_ → P_2_O_5(sln,700)_ | **ΔH_2_ = -164.60 ± 0.85 ^$^** |
| H_2_O_(l,25)_ → H_2_O_(g,700)_ | **ΔH_3_ = 69.0^*^** |
| 2 RE_(s, 25)_ + 3/2 O_2(g, 25)_ → RE_2_O_3(s, 25)_ | **ΔH_4_ ^$^** |
| 2 P_(s, 25)_ + 5/2 O_2(g, 25)_ → P_2_O_5(s, 25)_ | **ΔH_5_ = -1504.9 ± 0.5 ^$^** |
| H_2(g, 25)_ + 1/2 O_2(g, 25)_ → H_2_O_(l, 25)_ | **ΔH_6_ = -285.8 ± 0.1 ^$^** |
| 1/2 RE_2_O_3(s,25)_ + 1/2 P_2_O_5(s,25)_ + *n* H_2_O_(l,25)_ → REPO_4_ · *n* H_2_O_(s,25)_ | **ΔH_f,ox_(rh,25)** |
| 1/2 RE_2_O_3(s,25)_ + 1/2 P_2_O_5(s,25)_ → REPO_4 (s,25)_ | **ΔH_f,ox_(m,25)** |
| RE_(s, 25)_ + P_(s, 25)_ + *n* H_2(g, 25)_ + (2 + *n*/2) O_2(g, 25)_ → REPO_4_ · *n* H_2_O_(s,25)_ | **ΔH_f,el_(rh,25)** |
| RE_(s, 25)_ + P_(s, 25)_ + 2 O_2(g, 25)_ → REPO_4(s,25)_ | **ΔH_f,el_(m,25)** |
| REPO_4_ · *n* H_2_O_(s,25)_ → REPO_4 (s,25)_ + *n* H_2_O_(l,25)_ | **ΔH_rh→m_** |
| **ΔH_f,ox_(rh,25) = -ΔH_ds_(rh,700) + 1/2 ΔH_1_ + 1/2 ΔH_2_ + *n* ΔH_3_** |  |
| **ΔH_f,ox_(m,25) = -ΔH_ds_(m,700) + 1/2 ΔH_1_ + 1/2 ΔH_2_** |  |
| **ΔH_f,el_(rh,25) = 1/2 ΔH_4_ + 1/2 ΔH_5_ + *n* ΔH_6_ + ΔH_f,ox_(rh,25)** |  |
| **ΔH_f,el_(m,25) = 1/2 ΔH_4_ + 1/2 ΔH_5_ + ΔH_f,ox_(m,25)** |  |
| **ΔH_r→m_ = ΔH_ds_(rh,700) – ΔH_ds_(m,700) – *n* ΔH_3_** |  |

^$^ (Cheng and Navrotsky, 2003; Navrotsky, 2014; Ushakov et al., 2001)

^*^ (Guo et al., 2015)

Table A1b. Thermodynamic cycle used for Ce

| **Reaction** | **Enthalpy, kJ mol^-1^** |
| --- | --- |
| CePO_4_ · *n* H_2_O_(s,25)_ + 1/4 O_2(g,700)_ → CeO_2(sln,700)_ + 1/2 P_2_O_5(sln,700)_ + *n* H_2_O_(g,700)_ | **ΔH_ds_(rh,700)** |
| CePO_4(s,25)_ + 1/4 O_2(g,700)_ → CeO_2(sln,700)_ + 1/2 P_2_O_5(sln,700)_ | **ΔH_ds_(m,700)** |
| CeO_2(s,25)_ → CeO_2(sln,700)_ | **ΔH_7_ = 74.37 ± 0.75 ^$^** |
| O_2(g,25)_ → O_2(g,700)_ | **ΔH_8_ = 21.74 ^$^** |
| Ce_(s,25)_ + O_2(g,25)_ → CeO_2(s,25)_ | **ΔH_9_ = -1088.7 ± 1.5 ^$^** |
| 2 Ce_(s,25)_ + 3/2 O_2(g,25)_ → Ce_2_O_3(s,25)_ | **ΔH_10_ = -1796.2 ± 8.4 ^$^** |
| 1/2 Ce_2_O_3(s,25)_ + 1/2 P_2_O_5(s,25)_ + *n* H_2_O_(l, 25)_ → CePO_4_ · *n* H_2_O_(s,25)_ | **ΔH_f,ox_(rh,25)** |
| 1/2 Ce_2_O_3(s,25)_ + 1/2 P_2_O_5(s,25)_ → CePO_4(s,25)_ | **ΔH_f,ox_(m,25)** |
| Ce_(s, 25)_ + P_(s, 25)_ + *n* H_2(g, 25)_ + (2 + *n*/2) O_2(g, 25)_ → CePO_4_ · *n* H_2_O_(s,25)_ | **ΔH_f,el_(rh,25)** |
| Ce_(s, 25)_ + P_(s, 25)_ + 2 O_2(g, 25)_ → CePO_4(s,25)_ | **ΔH_f,el_(m,25)** |
| CePO_4_ · *n* H_2_O_(s,25)_ → CePO_4 (s,25)_ + *n* H_2_O_(l,25)_ | **ΔH_rh→m_** |
| **ΔH_f,ox_(rh,25) = -ΔH_ds_(rh,700) + ΔH_7_ + 1/2 ΔH_2_ - 1/4 ΔH_8_ + *n* ΔH_3_ + ΔH_9_ - 1/2 ΔH_10_** | |
| **ΔH_f,ox_(m,25) = -ΔH_ds_(m,700) + ΔH_7_ + 1/2 ΔH_2_ - 1/4 ΔH_8_ + ΔH_9_ - 1/2 ΔH_10_** |  |
| **ΔH_f,el_(rh,25) = 1/2 ΔH_10_ + 1/2 ΔH_5_ + *n* ΔH_6_ + ΔH_f,ox_(rh,25)** |  |
| **ΔH_f,el_(m,25) = 1/2 ΔH_10_ + 1/2 ΔH_5_ + ΔH_f,ox_(m,25)** |  |
| **ΔH_r→m_ = ΔH_ds_(rh,700) – ΔH_ds_(m,700) – *n* ΔH_3_** |  |

^$^ (Cheng and Navrotsky, 2003; Navrotsky, 2014; Ushakov et al., 2001)

Table A1c. Thermodynamic cycle used for Pr

| **Reaction** | **Enthalpy, kJ mol^-1^** |
| --- | --- |
| PrPO_4_ · *n* H_2_O_(s,25)_ → 1/2 Pr_2_O_3(sln,800)_ + 1/2 P_2_O_5(sln,800)_ + *n* H_2_O_(g, 800)_ | **ΔH_ds_(rh,800)** |
| PrPO_4(s,25)_ → 1/2 Pr_2_O_3(sln,800)_ + 1/2 P_2_O_5(sln,800)_ | **ΔH_ds_(m,800)** |
| Pr_2_O_3(s,25)_ → Pr_2_O_3(sln,800)_ | **ΔH_11_ = 16.50 ± 0.2 ^$^** |
| P_2_O_5(s,25)_ → P_2_O_5(sln,800)_ | **ΔH_12_ = -342.10 ± 7.9 ^&^** |
| H_2_O_(l,25)_ → H_2_O_(g,800)_ | **ΔH_13_ = 73.2 ^*^** |
| 2 Pr_(s, 25)_ + 3/2 O_2(g, 25)_ → Pr_2_O_3(s, 25)_ | **ΔH_14_ = -1809.6 ± 6.7 ^$^** |
| 1/2 Pr_2_O_3(s,25)_ + 1/2 P_2_O_5(s,25)_ + *n* H_2_O_(l,25)_ → PrPO_4_ · *n* H_2_O_(s,25)_ | **ΔH_f,ox_(rh,25)** |
| 1/2 Pr_2_O_3(s,25)_ + 1/2 P_2_O_5(s,25)_ → PrPO_4(s,25)_ | **ΔH_f,ox_(m,25)** |
| Pr_(s, 25)_ + P_(s, 25)_ + *n* H_2(g, 25)_ + (2 + *n*/2) O_2(g, 25)_ → PrPO_4_ · *n* H_2_O_(s,25)_ | **ΔH_f,el_(rh,25)** |
| Pr_(s, 25)_ + P_(s, 25)_ + 2 O_2(g, 25)_ → REPO_4(s,25)_ | **ΔH_f,el_(m,25)** |
| PrPO_4_ · *n* H_2_O_(s,25)_ → PrPO_4(s,25)_ + *n* H_2_O_(l,25)_ | **ΔH_rh→m_** |
| **ΔH_f,ox_(rh,25) = -ΔH_ds_(rh,800) + 1/2 ΔH_11_ + 1/2 ΔH_12_ + *n* ΔH_13_** |  |
| **ΔH_f,ox_(m,25) = -ΔH_ds_(m,800) + 1/2 ΔH_11_ + 1/2 ΔH_12_** |  |
| **ΔH_f,el_(rh,25) = 1/2 ΔH_14_ + 1/2 ΔH_5_ + *n* ΔH_6_ + ΔH_f,ox_(rh,25)** |  |
| **ΔH_f,el_(m,25) = 1/2 ΔH_14_ + 1/2 ΔH_5_ + ΔH_f,ox_(m,25)** |  |
| **ΔH_r→m_ = ΔH_ds_(rh,800) – ΔH_ds_(m,800) – *n* ΔH_13_** |  |

^$^ (Cheng and Navrotsky, 2003; Navrotsky, 2014; Ushakov et al., 2001) ;

^*^ (Guo et al., 2015) ;

^&^ (Popa et al., 2008)

Cheng, J., and Navrotsky, A. (2003). Enthalpies of formation of LaBO_3_ perovskites (B = Al, Ga, Sc, and In). *J. Mater. Res.* 18, 2501–2508. doi:10.1557/JMR.2003.0348.

Guo, X., Szenknect, S., Mesbah, A., Labs, S., Clavier, N., Poinssot, C., et al. (2015). Thermodynamics of formation of coffinite, USiO_4_. *Proc. Natl. Acad. Sci.* 112, 6551–6555. doi:10.1073/pnas.1507441112.

Navrotsky, A. (2014). Progress and new directions in calorimetry: a 2014 perspective. *J. Am. Ceram. Soc.* 97, 3349–3359. doi:10.1111/jace.13278.

Popa, K., Shvareva, T., Mazeina, L., Colineau, E., Wastin, F., Konings, R. J. M., et al. (2008). Thermodynamic properties of CaTh(PO4)2 synthetic cheralite. *Am. Mineral.* 93, 1356–1362. doi:10.2138/am.2008.2794.

Ushakov, S. V., Helean, K. B., Navrotsky, A., and Boatner, L. A. (2001). Thermochemistry of rare-earth orthophosphates. *J. Mater. Res.* 16, 2623–2633. doi:10.1557/JMR.2001.0361.

Table A2. Thermodynamic data used for calculations (numbers in brackets are references to literature and formulas)

| **ELEMENTS** | **ΔG_(f,el)_, kJ/mol** | **ΔH_(f,el)_, kJ/mol** | **ΔS_(f,el)_, J/mol K** | **ΔG_(f,ox)_, kJ/mol** | **ΔH_(f,ox)_, kJ/mol** | **ΔS_(f,ox)_, J/mol K** | **S^°^_m_, J/mol K** |
| --- | --- | --- | --- | --- | --- | --- | --- |
| La | - | - | - | - | - | - | 56.9 ± 2.5 [1] |
| Ce | - | - | - | - | - | - | 69.5 ± 8.4 [1] |
| Pr | - | - | - | - | - | - | 73.9 ± 4.2 [1] |
| Nd | - | - | - | - | - | - | 71.1 ± 4.2 [1] |
| Sm | - | - | - | - | - | - | 69.5 ± 2.1 [1] |
| Eu | - | - | - | - | - | - | 80.8 ± 0.2 [1] |
| Gd | - | - | - | - | - | - | 68.5 ± 1.3 [1] |
| O_2_(g) | - | - | - | - | - | - | 205.2 ± 0.1 [1] |
| H_2_(g) | - | - | - | - | - | - | 130.7 ± 0.1 [1] |
| P(cr) | - | - | - | - | - | - | 41.1 ± 0.3 [1] |
| **OXIDES** | **ΔG_(f,el)_, kJ/mol** | **ΔH_(f,el)_, kJ/mol** | **ΔS_(f,el)_, J/mol K** | **ΔG_(f,ox)_, kJ/mol** | **ΔH_(f,ox)_, kJ/mol** | **ΔS_(f,ox)_, J/mol K** | **S^°^_m_, J/mol K** |
| La_2_O_3_ | -1703.9 ± 3.6 [2] | -1791.6 ± 1.6 [2] | -294.1 ± 2.6 [2] | - | - | - | 127.32 ± 0.4 [2] |
| Ce_2_O_3_ | -1709.6 ± 10.7 [2] | -1799.8 ± 8.4 [2] | -302.6 ± 8.9 [2] | - | - | - | 148.10 ± 0.3 [2] |
| Pr_2_O_3_ | -1719.6 ± 7.6 [2] | -1809.9 ± 3.3 [2] | -302.8 ± 4.6 [2] | - | - | - | 152.70 ± 0.3 [2] |
| Nd_2_O_3_ | -1719.7 ± 6.6 [2] | -1806.9 ± 3.0 [2] | -292.4 ± 3.6 [2] | - | - | - | 158.70 ± 1.0 [2] |
| Sm_2_O_3_ | -1734.8 ± 5.0 [2] | -1823.0 ± 4.0 [2] | -295.7 [2] | - | - | - | 150.60 ± 0.3 [2] |
| Eu_2_O_3_ | -1553.6 ± 8.8 [2] | -1650.4 ± 4.0 [2] | -324.6 ± 4.8 [2] | - | - | - | 135.40 ± 2.0 [2] |
| Gd_2_O_3_ | -1732.4 ± 5.0 [2] | -1819.7 ± 8.4 [2] | -292.8 [2] | - | - | - | 152.90 ± 0.2 [2] |
| H_2_O | -237.1 ± 0.1 [2] | - | - | - | - | - | 69.95 ± 0.1 [1] |
| P_2_O_5_ | -1361.6 ± 0.5 [2] | - | - | - | - | - | 114.4 ± 0.4 [1] |
| **MONAZITES** | **ΔG_(f,el)_, kJ/mol** | **ΔH_(f,el)_, kJ/mol** | **ΔS_(f,el)_, J/mol K** | **ΔG_(f,ox)_, kJ/mol** | **ΔH_(f,ox)_, kJ/mol** | **ΔS_(f,ox)_, J/mol K** | **S^°^_m_, J/mol K** |
| LaPO_4_ | -1874.6 ± 6.2 [2] | -1994.4 ± 4.3 | -401.7 ± 7.5 | -341.8 ± 6.7 | -346.11 ± 4.15 | -14.3 ± 7.9 | 107.1 ± 7.9 |
| CePO_4_ | -1845.0 ± 8.2 [2] | -1963.8 ± 9.4 | -398.5 ± 12.5 | -309.4 ± 11.2 | -311.45 ± 7.21 | -6.8 ± 13.3 | 125.0 ± 13.3 |
| PrPO_4_ | -1859.7 ± 8.6 [2] | -1983.5 ± 6.3 | -415.3 ± 10.6 | -319.1 ± 10.1 | -326.11 ± 5.90 | -23.5 ± 11.7 | 110.6 ± 11.7 |
| NdPO_4_ | -1844.9 ± 6.4 [2] | -1964.7 ± 5.1 | -401.9 ± 8.1 | -304.2 ± 7.9 | -308.78 ± 4.60 | -15.3 ± 9.1 | 121.8 ± 9.2 |
| SmPO_4_ | -1846.9 ± 10.2 [2] | -1965.7 ± 5.3 | -398.5 ± 11.5 | -298.7 ± 10.8 | -301.77 ± 4.47 | -10.3 ± 11.7 | 122.7 ± 11.7 |
| EuPO_4_ | -1744.6 ± 13.2 [2] | -1870.6 ± 4.9 | -422.5 ± 14.4 | -287.0 ± 14.6 | -286.90 ± 4.13 | 0.4 ± 15.1 | 125.9 ± 15.2 |
| GdPO_4_ | -1838.3 ± 5.4 [2] | -1956.1 ± 7.2 | -395.0 ± 8.9 | -291.3 ± 6.4 | -293.80 ± 3.97 | -8.3 ± 7.6 | 125.9 ± 7.6 |

Table A2. Continued

| **RHABDOPHANES** | **ΔG_(f,el)_, kJ/mol** | **ΔH_(f,el)_, kJ/mol** | **ΔS_(f,el)_, J/mol K** | **ΔG_(f,ox)_, kJ/mol** | **ΔH_(f,ox)_, kJ/mol** | **ΔS_(f,ox)_, J/mol K** | **S^°^_m_, J/mol K** |
| --- | --- | --- | --- | --- | --- | --- | --- |
| LaPO_4_ · 0.804 H_2_O | -2036 ± 6 [3] | -2220.9 ± 4.5 | -618.7 ± 7.4 | -313.1 ± 6.4 | -342.92 ± 4.29 | -100.0 ± 7.7 | 77.6 ± 7.8 |
| CePO_4_ · 0.732 H_2_O | -2012 ± 8 [3] | -2189.7 ± 9.4 | -594.9 ± 12.2 | -303.3 ± 10.9 | -328.23 ± 7.21 | -83.7 ± 13.1 | 99.3 ± 13.1 |
| PrPO_4_ · 0.709 H_2_O | -2013 ± 8 [3] | -2181.7 ± 6.4 | -565.6 ± 10.2 | -304.2 ± 9.6 | -321.54 ± 6.05 | -58.0 ± 11.3 | 125.7 ± 11.3 |
| NdPO_4_ · 0.746 H_2_O | -2013 ± 6 [3] | -2178.7 ± 5.1 | -556.7 ± 7.8 | -295.2 ± 7.5 | -309.60 ± 4.57 | -48.3 ± 8.8 | 141.0 ± 8.8 |
| SmPO_4_ · 0.636 H_2_O | -1982 ± 10 [3] | -2156.8 ± 5.1 | -587.6 ± 11.1 | -282.6 ± 10.5 | -311.13 ± 4.22 | -95.6 ± 11.3 | 82.0 ± 11.3 |
| EuPO_4_ · 0.555 H_2_O | -1869 ± 13 [3] | -2042.4 ± 5.9 | -580.3 ± 14.2 | -280.2 ± 14.3 | -300.15 ± 4.07 | -66.8 ± 14.9 | 97.5 ± 15.0 |
| GdPO_4_ · 0.533 H_2_O | -1952 ± 5 [3] | -2119.1 ± 7.2 | -559.7 ± 8.7 | -278.8 ± 6.1 | -304.45 ± 3.96 | -85.9 ± 7.2 | 85.6 ± 7.2 |
| **REACTION** | | | | | **ΔH_reaction_, kJ/mol** | **ΔS_reaction_, J/mol K** | **ΔG_reaction_, kJ/mol** |
| LaPO_4_ · 0.804 H_2_O → LaPO_4_ + 0.804 H_2_O | | | | | -3.19 ± 1.58 | 85.7 ± 11.1 | -28.8 ± 8.5 |
| CePO_4_ · 0.732 H_2_O → CePO_4_ + 0.732 H_2_O | | | | | 16.79 ± 1.18 | 76.9 ± 18.7 | -6.2 ± 14.4 |
| PrPO_4_ · 0.709 H_2_O → PrPO_4_ + 0.709 H_2_O | | | | | -4.57 ± 2.99 | 34.5 ± 16.3 | -14.9 ± 12.3 |
| NdPO_4_ · 0.746 H_2_O → NdPO_4_ + 0.746 H_2_O | | | | | 0.82 ± 1.80 | 33.0 ± 12.7 | -9.1 ± 9.5 |
| SmPO_4_ · 0.636 H_2_O → SmPO_4_ + 0.636 H_2_O | | | | | 9.36 ± 1.61 | 85.2 ± 16.3 | -20.1 ± 10.8 |
| EuPO_4_ · 0.555 H_2_O → EuPO_4_ + 0.555 H_2_O | | | | | 13.25 ± 1.47 | 67.2 ± 21.3 | -9.8 ± 13.1 |
| GdPO_4_ · 0.533 H_2_O → GdPO_4_ + 0.533 H_2_O | | | | | 10.65 ± 1.33 | 77.6 ± 10.5 | -12.5 ± 8.0 |

All errors of calculated values are propagated.

[1] Thermodynamic properties of minerals and related substances at 298.15 K and 1 Bar (105 Pascals) pressure and at higher temperatures, Robie and Hemingway (1979)

[2] Thermodynamics of solid phases containing rare earth oxides, Navrotsky et al. (2015)

[3] Determination of the solubility of rhabdophanes LnPO_4_ · 0.667 H_2_O (Ln = La to Dy), Gausse et al. (2016)
